# Comment on Korkmaz et al. A Deep Learning and Explainable AI-Based Approach for the Classification of Discomycetes Species. *Biology* 2025, *14*, 719

**DOI:** 10.3390/biology15020106

**Published:** 2026-01-06

**Authors:** Emmanuel Pio Pastore

**Affiliations:** Department of Biology, Ecology and Earth Science, University of Calabria, 87036 Rende, Italy; pstmnl03e19c349r@studenti.unical.it

To the Editor: Korkmaz et al. report high performance for macrofungi identification using convolutional networks with Grad-CAM and Score-CAM visualizations, trained on a 2800-image compilation from natural photographs and GBIF, split 60/20/20 into training, validation, and testing [1]. The topic is timely; nevertheless, some choices can overstate accuracy and make transfer to real-world surveys less certain.

First, independence between training and testing depends on how images relate to specimens, photographers, and sites. When multiple shots of the same individual or collection event are randomly dispersed across the split, near-duplicates and shared backgrounds may leak scene-level cues into evaluation. Grouping by specimen, collector, location, or photographer and then withholding groups—rather than individual images—would better reflect deployment, in line with cross-validation strategies for spatial or hierarchical data [2].

Second, preprocessing, augmentation, and any feature selection should be restricted to the training folds and then applied unchanged to held-out data. Using the full dataset to set parameters, or optimizing on the validation set without nesting, allows target-related structure to seep into assessment, inflating discrimination. A fully nested workflow (split, fit transforms and tuning only on training, freeze, then evaluate) limits selection bias and yields estimates closer to field performance [2].

Third, very high accuracy can arise from shortcuts: models may latch onto background texture, color casts, or photographic conventions instead of morphology, especially with mixed sources. Saliency maps help, but qualitative overlays alone are not decisive; sanity checks and quantitative focus tests can verify that explanations change with the model and that highlighted regions matter for the prediction [3,4]. In addition, calibrated probabilities with reliability assessment—rather than raw scores—let teams choose thresholds appropriate for biodiversity monitoring and curation workflows [5].

For taxonomy and conservation work, what endures is performance on new cameras, unseen habitats, and independent collections. Organizing the split at the level of specimens or sources, nesting all preprocessing and tuning, adding external or site-withheld evaluation, and reporting calibration would make the reported figures a dependable guide for practitioners while preserving the useful interpretability provided by XAI.
